# Validation of T2* in-line analysis for tissue iron quantification at 1.5 T

**DOI:** 10.1186/s12968-016-0243-4

**Published:** 2016-04-27

**Authors:** Mohammed H. Alam, Taigang He, Dominique Auger, Gillian C. Smith, Peter Drivas, Rick Wage, Cemil Izgi, Karen Symmonds, Andreas Greiser, Bruce S. Spottiswoode, Lisa Anderson, David Firmin, Dudley J. Pennell

**Affiliations:** Cardiovascular Biomedical Research Unit, Royal Brompton Hospital, Sydney Street, London, SW3 6NP UK; National Heart and Lung Institute, Imperial College, London, UK; Cardiovascular Science Research Center, St George’s, University of London, London, UK; Siemens AG Healthcare, Erlangen, Germany; Siemens Medical Solutions USA Inc, Malvern, PA USA

**Keywords:** CMR, T2*, In-line processing, Heart, Liver, Iron overload

## Abstract

**Background:**

There is a need for improved worldwide access to tissue iron quantification using T2* cardiovascular magnetic resonance (CMR). One route to facilitate this would be simple in-line T2* analysis widely available on MR scanners. We therefore compared our clinically validated and established T2* method at Royal Brompton Hospital (RBH T2*) against a novel work-in-progress (WIP) sequence with in-line T2* measurement from Siemens (WIP T2*).

**Methods:**

Healthy volunteers (*n* = 22) and patients with iron overload (*n* = 78) were recruited (53 males, median age 34 years). A 1.5 T study (Magnetom Avanto, Siemens) was performed on all subjects. The same mid-ventricular short axis cardiac slice and transaxial slice through the liver were used to acquire both RBH T2* images and WIP T2* maps for each participant. Cardiac white blood (WB) and black blood (BB) sequences were acquired. Intraobserver, interobserver and interstudy reproducibility were measured on the same data from a subset of 20 participants.

**Results:**

Liver T2* values ranged from 0.8 to 35.7 ms (median 5.1 ms) and cardiac T2* values from 6.0 to 52.3 ms (median 31 ms). The coefficient of variance (CoV) values for direct comparison of T2* values by RBH and WIP were 6.1–7.8 % across techniques. Accurate delineation of the septum was difficult on some WIP T2* maps due to artefacts. The inability to manually correct for noise by truncation of erroneous later echo times led to some overestimation of T2* using WIP T2* compared with the RBH T2*. Reproducibility CoV results for RBH T2* ranged from 1.5 to 5.7 % which were better than the reproducibility of WIP T2* values of 4.1–16.6 %.

**Conclusions:**

Iron estimation using the T2* CMR sequence in combination with Siemens’ in-line data processing is generally satisfactory and may help facilitate global access to tissue iron assessment. The current automated T2* map technique is less good for tissue iron assessment with noisy data at low T2* values.

## Background

Accurate and robust measurement of myocardial iron stores is essential for preventing cardiac disease and managing chelation treatment in patients with thalassemia, sickle cell disease, aplastic anemia, myelodysplasia, and other transfusion-dependant anemias [1–3]. T2* cardiovascular magnetic resonance (CMR) is effective and robust for tissue iron measurement in myocardial siderosis [4–8]; it is non-invasive and now considered the method of choice for myocardial and liver iron quantification [1, 9]. The underlying mechanism is that MR relaxation time of hydrogen nuclei falls with increasing amounts of storage iron. In recent studies, it has been demonstrated that tissue iron is the dominant determinant of myocardial relaxation, indicating that T2* CMR can measure the iron content accurately [10, 11].

The development of T2* CMR has made a great impact on patient management and survival, as no alternative method (e.g., serum ferritin, myocardial biopsy, liver biopsy or susceptometry) gives reliable information about cardiac iron loading. In the UK where it was first introduced clinically, >70 % reductions in cardiac mortality for thalassemia major (TM) have been documented [12]. However, barriers to using T2* CMR exist. Despite the successful T2* development, a recent international survey [13] indicates that the adoption of this technique is heterogeneous and that cases of high liver/myocardial iron concentration are still abundant, especially in developing countries where most of the patients are located. Apart from sequence and practical imaging issues, one particular challenge is that a post-processing step of T2* measurement using special software and training is required, resulting in some centers being unable to offer this lifesaving technique on a routine basis, or using locally developed software which has not been validated.

After a decade of development, the single breath-hold multi-echo gradient CMR T2* sequence is available from the major vendors, including Siemens, Philips and GE. One recent approach to further increasing T2* access is the vendors’ attempt to develop an in-line processing algorithm to generate T2* maps immediately after the scan. This obviates the need for third-party software, with users being able to simply draw a region-of-interest (ROI) on the map generated in the scanner console. This may streamline clinical workflow with the additional advantage of decreased user interaction. One such product is provided by Siemens Healthcare (Erlangen, Germany), named the Works in Progress T2* package (WIP). However, there are little validation data on this type of potentially useful product. In this study therefore, we aimed to directly compare WIP T2* with the established T2* technique at Royal Brompton Hospital (RBH) on a population of healthy volunteers and patients with iron overload conditions.

## Methods

### Patient population

A total of 22 healthy volunteers and 78 patients were recruited (*N* = 100, 53 males, aged 13 to 81 years, median 34 years). Among patients referred for routine iron assessment, there were 39 with beta-thalassaemia major, 15 with sickle cell disease, 10 with hereditary hemochromatosis, and 14 with other iron overload conditions. The study was approved by the North-West Thames Ethics Committee. All participants gave written informed consent.

### MR protocols

All participants were scanned on a 1.5 T scanner (Magnetom Avanto, Siemens Healthcare, Erlangen, Germany) equipped with high performance gradients and a six-element cardiac phased array coil and a spine array coil. The same mid-ventricular short axis cardiac slice and transaxial slice through the liver were used for both RBH T2* images (developed in-house based on the Siemens CV sequence) and WIP T2* (Siemens WIP 448, VB17) maps for each participant. Cardiac white blood (WB) and black blood (BB) and liver sequences [14, 15] were all acquired for every patient. For RBH and WIP T2* sequences, major parameters were set to be identical or very close as optimised for each participant individually, as previously described [14, 15] and according to our clinical practice.

For the heart, a single 10 mm mid-ventricular short axis slice was imaged at eight echo-times (ranging from 1.5 ms to 16.9 ms, increment 2.18 ms). The BB sequences used double inversion recovery pulses to suppress the blood signal and data were acquired every other cardiac cycle, which made the effective sequence two cardiac cycles long. For the liver, a mid-liver transverse slice was acquired positioned to cover both lobes using the sequence optimised for liver iron load with the following acquisition parameters: flip angle of 20°, repetition time of 200 ms, 12 echo times (0.93, 2.27, 3.61, 4.95, 6.29, 7.63, 8.97, 10.4, 11.8, 13.2, 14.6, 16 ms), slice thickness of 10 mm. Fat saturation was applied and the liver images were acquired within a breath-hold of approximately 13 s.

### T2*measurement

For all cardiac analyses, a homogeneous full thickness ROI was drawn in the septum. Care was taken to exclude epicardial structures and blood pool from the contours. For all liver analyses, three large ROIs (each >3 cm^2^) were drawn in homogenous areas of liver parenchyma, excluding blood vessels.

For RBH T2* measurement, the mean signal intensity of ROIs was measured in the series of increasing TE images, and the data were plotted against each TE to form an exponential decay curve. Truncation was used to account for the plateau observed due to low signal to noise ratio (SNR) at the later echo times. All T2* measurements were carried out on a personal computer using Thalassemia-Tools, a plug-in of CMRtools (Cardiovascular Imaging Solutions, London, UK). For WIP T2* measurement, like-for-like ROIs were directly drawn on the magnitude images and copied to the T2* maps generated on the scanner and the mean ROI values were calculated for T2*. This was performed because of the difficulty of confidently identifying the myocardium on some T2* maps.

In order to evaluate the intra and inter-observer reproducibility, all in-vivo data (*n* = 20) were ordered randomly. For the intra-observer reproducibility, all selected data were blindly reanalyzed by the same operator after 15 days. For the interobserver reproducibility, the data were analyzed by two independent operators blinded to each other’s T2* measurement. For interstudy reproducibility, repeated scans performed on the same day, were analyzed by the same observer blindly.

Artefact scoring was performed using a 6 point scale as previously described [16]. In brief, the analysis was conducted using the following scale: 0- Very poor image quality with unusable images; 1- Poor image quality, just able to make out the heart/liver but a lot of artefact; 2- Average image quality, not all of septum/liver clearly seen and a lot of artefact; 3- Good image quality with moderate septal/liver artefact; 4- Very good quality, with minimal septal/liver artefacts; 5- Excellent image quality with no significant septal/liver artefact. Higher scores indicated fewer artefacts with higher image quality.

### Statistics

Reproducibility analyses were assessed using the mean and standard deviation (SD) of the differences as described by the Bland-Altman analysis. The coefficient of variation (CoV), defined as the SD of the differences between two independent measurements divided by their means and expressed as a percentage, was used to assess the intra- and inter-observer and inter-study variability. Summary data were expressed using the mean or median for normal and non-normal data respectively. Correlation analysis was performed using Pearson’s test. *R*^*2*^ was used to describe how well the exponential model fitted the empirical data. Wilcoxon signed rank test was used for artefact score comparison. A *p* value of <0.05 was considered statistically significant.

## Results

**Fig. 1:**
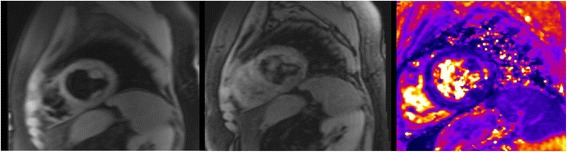
Typical short axis mid-ventricular slices obtained from a thalassemia patient (left- RBH T2* sequence; middle- WIP sequence; right- corresponding in-line T2* map)

All participants underwent scans without complications and all scans were successfully analyzed. No data was excluded from analysis. Liver T2* values ranged from 0.8 ms to 35.7 ms (median 5.1 ms) and cardiac T2* values from 6.0 ms to 52.3 ms (median 31 ms), both representing a wide spectrum of tissue iron loading conditions. Representative images acquired from a TM patient of the same mid-ventricular slice for RBH T2* and WIP T2* are shown in Fig. 1. When considering all participant images, four issues were noted: (A) all images of both RBH and WIP T2* were diagnostic; (B) there were generally more artefacts in WIP T2* maps than on the RBH T2* images, which was attributable to the consequences of the fitting; (C) delineation of the septum was sometimes difficult in cardiac WIP T2* maps due to poor contrast and artefacts; (D) in severely iron loaded cases, there were “black holes” in some liver WIP T2* maps, meaning that the mapping algorithm failed to produce reasonable T2* values for these pixels.

### Relation between RBH and WIP T2*measurements

**Fig. 2:**
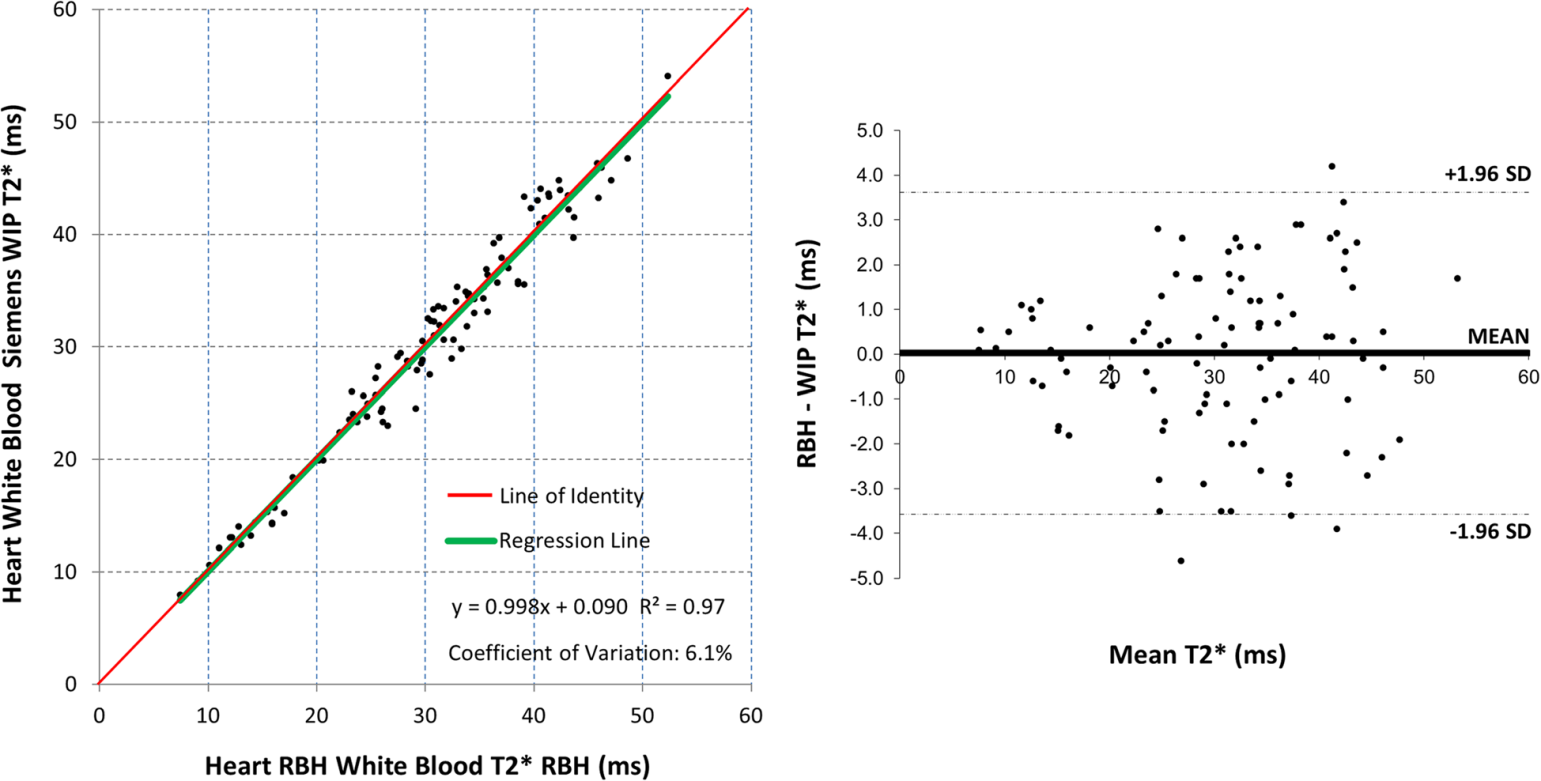
Scatter plot with the line of identity (*left*) and Bland-Altman plot (*right*) showing the comparison for the white blood RBH and WIP T2* cardiac sequences. The 95 % confidence intervals are shown as dotted lines on the Bland-Altman plots

**Fig. 3:**
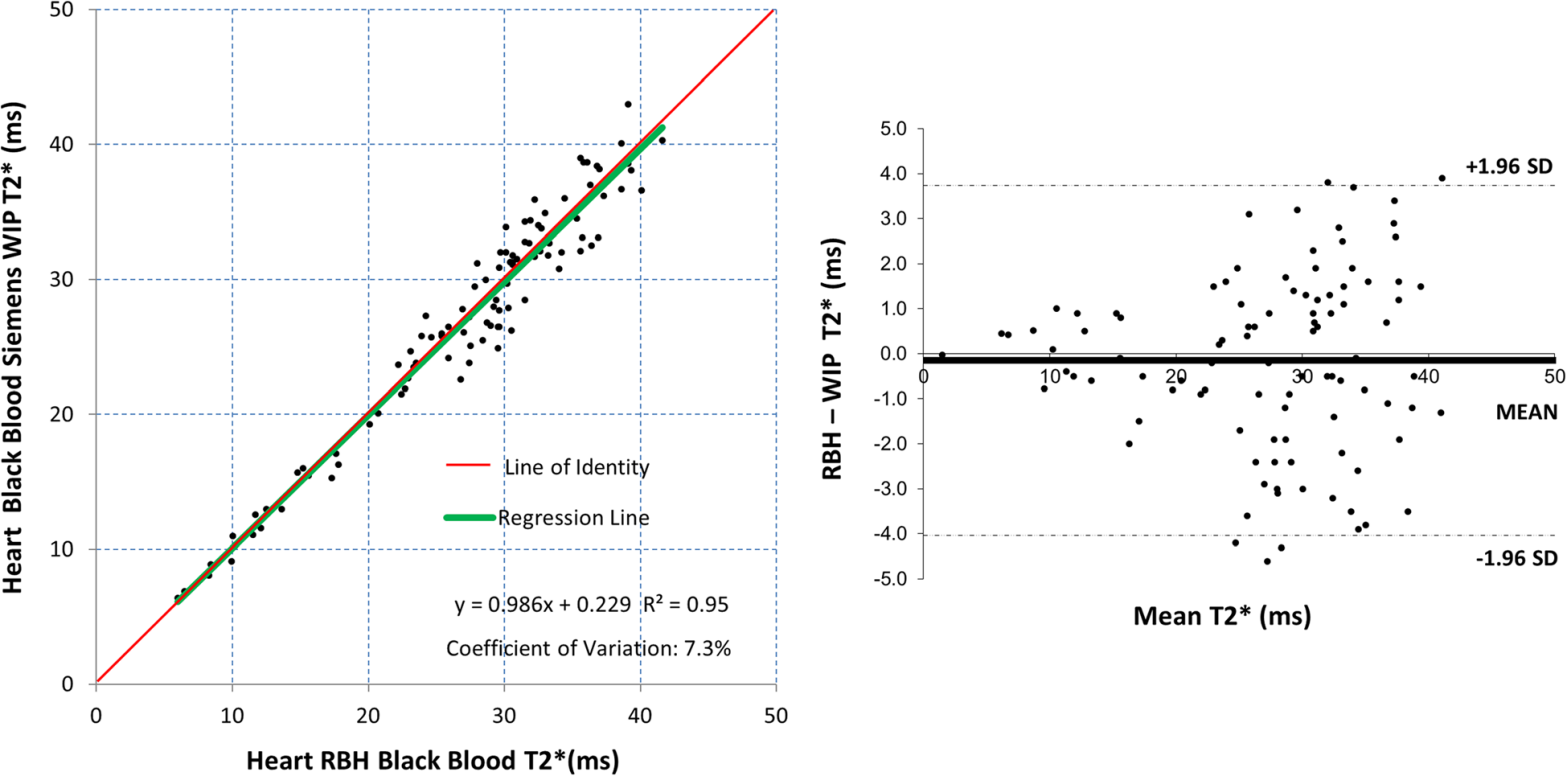
Scatter plot with the line of identity (*left*) and Bland-Altman plot (*right*) showing the comparison for the black blood RBH and WIP T2* cardiac sequences. The 95 % confidence intervals are shown as dotted lines on the Bland-Altman plots

**Fig. 4:**
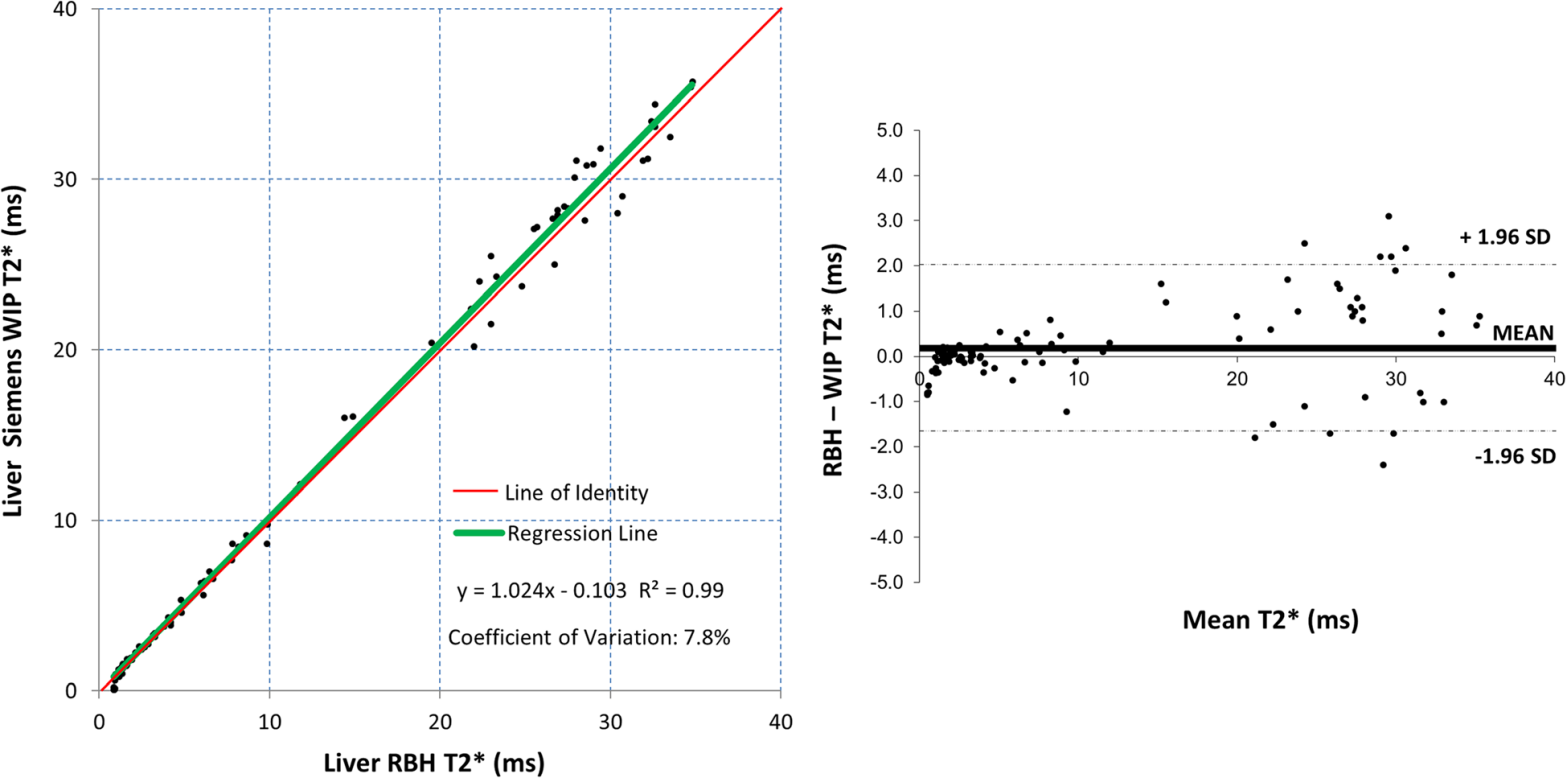
Scatter plot with the line of identity (*left*) and Bland-Altman plot (*right*) showing the comparison for the RBH and WIP T2* liver sequences. The 95 % confidence intervals are shown as dotted lines on the Bland-Altman plots

For heart WB T2*, there was a linear correlation (Fig. 2, *R*^*2*^ = 0.97, CoV = 6.1 %) between RBH and WIP techniques. For heart BB T2*, the correlation is shown in Fig. 3 (*R*^*2*^ = 0.95, CoV = 7.3 %). For liver T2*, the linear correlation between RBH and WIP T2* techniques is shown in Fig. 4 (*R*^*2*^ = 0.99, CoV = 7.8 %).

### Interobserver, intraobserver, and interstudy reproducibility

**Fig. 5:**
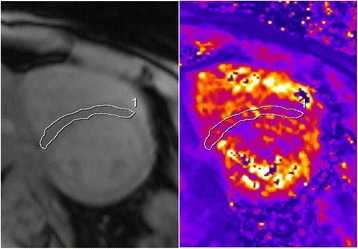
Example showing that the placing of an ROI in the septum was generally straightforward using the magnitude images (*left*), but sometimes difficult on the WIP T2* map (*right*)

The results of the reproducibility studies are summarized in Table 1. For RBH T2*, the reproducibility was good with the CoV ranging from 1.5 to 5.7 % which compared with WIP T2* values which were less good, being 4.1–16.6 %. An example of the difficulty in confidently delineating the septum on the WIP T2* map is shown in Fig. 5.

**Table 1:** Reproducibility data

Sequence	Intraobserver	Interobserver	Interstudy	Artefact score
	CoV (%)	CoV (%)	CoV (%)	(mean)
	RBH/WIP	RBH/WIP	RBH/WIP	RBH/WIP
1.5 T Heart White blood	3.2/5.1	3.3/10.4	5.5/9.3	4.25/3.27
1.5 T Heart Black blood	1.5/4.1	2.8/10.1	4.6/7.5	4.39/4.24
1.5 T Liver	2.0/6.1	4.2/16.6	5.7/10.2	4.06/4.06

*CoV* coefficient of variance, *RBH* Royal Brompton Hospital, *WIP* work-in-progress

### Artefact scoring

The artefact scores are shown in Table 1. There were more artefacts with the white blood heart sequence, compared to the black blood sequence (*p* < 0.001). There were also some differences between the WIP and RBH techniques in artefact scores, with RBH better for the white blood heart sequence (*p* < 0.001).

## Discussion

There is a need for improved access to T2* CMR and more effort is needed to achieve this goal. Among these efforts, a dedicated T2* sequence combined with in-line processing has the promise to simplify T2* analysis and enable confident T2* measurement in less experienced centers. In this validation study, comparison of the RBH T2* values (being used as a gold-standard) and the WIP T2* values at 1.5 T showed good general agreement for heart and liver with clinically acceptable variances of 6.1 to 7.8 %. Our data suggest that the automated WIP technique may prove useful in clinical practice.

Reproducibility of the RBH T2* and WIP techniques showed that the RBH T2* analyses had reproducibility that was in the region of 2-fold superior (Table 1). The image quality of WIP T2* was generally good and this suggests that the performance of the automated in-line processing algorithm was probably suboptimal. We attribute this mainly to the problems encountered in some cases in correctly specifying the full thickness septum ROI on the T2* WIP map, as illustrated in Fig. 5. The artefact scores were usually higher for the RBH T2* technique indicating fewer artefacts with the RBH technique, but the differences overall were small. An issue which affected comparability was the “black holes” which occurred in some T2* maps with low T2* values, where the WIP analysis algorithm failed to assign a T2* value to the associated pixels and assigned a zero value, which led to a reduced T2* for the ROI. These issues may be addressed for improved performance in future releases of the WIP technique. Nevertheless, according to the Bland-Altman analyses, agreement between WIP techniques and gold-standard RBH sequence seemed highest in the ranges of particular clinical interest when myocardial T2* was below 20 ms and in liver T2* was below 5 ms.

There have been considerable efforts in parameter mapping development at the pixel level for tissue characterisation using CMR [17, 18]. The challenge is that there is no in-vivo gold standard for this pixel-wise approach due to the problem of motion [19, 20]. For this reason, the validation itself is often reduced to a conventional ROI based method, namely a ROI drawn in the map instead of a real pixel-wise validation. For tissue iron quantification using T2*, particular caution should be taken for clinical use of this pixel-wise approach. First, with inherently low SNR images compounded by the well-known Rician noise distribution, the pixel-wise T2* measurement error can amount to 150 % for the unprocessed data [18]. Second, although using an ROI on a pixel map can reduce the measurement error, this approach has not been scientifically justified. In brief, a pixel-wise mapping techniques are theoretically appealing, but meaningful measurements can only be made if the map is accurate.

There are limitations to this study. Cardiac T2* measurements were based on imaging and analysing the septum, as myocardial T2* can only be measured reliably in this region due to susceptibility artefacts. However, mid-ventricular septal T2* is highly representative of global iron concentration [21], and there is no significant segmental variation of iron deposition across the heart [21]. For the liver, it is generally accepted that iron distribution is relatively homogeneous, but we only measured T2* in a small region. Another limitation is that only a single vendor of Siemens was selected in this study. A future study of multi-vendor validation is warranted.

## Conclusion

There have been significant technical advances in T2* CMR over recent years and it is now the method of choice for cardiac and hepatic iron quantification in clinical practice. T2* sequence in combination with vendor’s inline data processing development has the promise to improve global access to tissue iron assessment. However, due to their inherent vulnerability to low SNR, current T2* mapping and inline analysis techniques are not yet fully optimised for tissue iron assessment. Further scientific development is needed to improve the mapping technique, which should be followed by validation in a large patient cohort.

## Abbreviations

BB: black blood
CMR: cardiovascular magnetic resonance
CoV: coefficient of variation
RBH: Royal Brompton Hospital
ROI: region of interest
SNR: signal to noise ratio
WB: white blood
WIP: work in progress
